# Proteinuria and Palmar Clues: Rediscovering the Renal Face of Syphilis

**DOI:** 10.7759/cureus.85031

**Published:** 2025-05-29

**Authors:** Alexis M Chrystman, Anvit Reddy, Yasasvhinie Santharam, Oshin Rai, Shiguang Liu, Ronald L Mars, Rafik Jacob

**Affiliations:** 1 Internal Medicine, University of Florida College of Medicine – Jacksonville, Jacksonville, USA; 2 Pathology and Laboratory Medicine, University of Florida College of Medicine – Jacksonville, Jacksonville, USA; 3 Nephrology, University of Florida College of Medicine – Jacksonville, Jacksonville, USA

**Keywords:** kidney biopsy, kidney involvement in syphilis, membranous nephropathy, rare presentation of nephrotic syndrome, secondary syphilis, skin punch biopsy

## Abstract

Syphilis is a sexually transmitted infection that remains prevalent in our population despite available curative treatment. Here, we present a case of a 35-year-old female with a presenting symptom of dry rash on multiple regions of her body. This rash was not initially identified as syphilis, and the patient’s disease progressed to involve the kidney. This systemic manifestation of secondary syphilis led to a membranous nephropathy (MN). Following treatment of the underlying infection, the patient’s symptoms and renal dysfunction fully resolved. This case highlights the wide-ranging impacts of syphilis as it continues to spread in our population, and that early recognition and targeted therapy can lead to favorable renal outcomes with a resolution of systemic manifestations.

## Introduction

Syphilis, a curable sexually transmitted disease caused by the bacterium *Treponema pallidum*, has infected humans for thousands of years. With the advent of penicillin by Alexander Fleming in 1928, the formerly deadly disease was nearly eradicated. However, this progress has not been maintained. Since 1990, there has been a rise in syphilis cases worldwide [1]. Historically, its prevalence was high in low- and middle-income countries, but the rates have increased in high-income countries [2]. In the United States, syphilis rates hit their lowest at 2.1 cases per 100,000 in the year 2000. Since then, the rates have increased to 15.6 cases per 100,000 males [1]. In low- and middle-income countries, the vertical transmission of syphilis still leads to major adverse pregnancy outcomes [2]. With the rise in syphilis rates, prior rare complications of advanced disease have risen as well.

Syphilis is unique in its stepwise progression. Primary syphilis often goes unrecognized due to its painless nature, a chancre at the inoculation site [2], while secondary syphilis has been referred to as “The Great Imitator” due to its many unique manifestations [3]. The most well-known exam findings include rash of the palms and soles, alongside generalized lymphadenopathy. Other physical exam findings include mucosal lesions or condyloma lata. Due to its disseminated nature and potential latent period, spirochetes can become embedded in several organs, including the liver, eyes, joints, or kidneys [2,3]. Patient-reported symptoms of secondary syphilis can thus be nonspecific, including fever, fatigue, muscle aches, joint pain, headache, and sore throat, leading to a difficult diagnosis. Symptoms of secondary syphilis typically resolve in less than 30 days, but some patients report symptoms lasting up to 240 days [4]. Finally, if syphilis is left untreated, it can progress to the tertiary phase. At this stage, it can invade the nervous system to cause a wide range of symptoms, including loss of balance, blindness, memory problems, personality changes, or seizures [3].

As noted above, one of the manifestations of secondary syphilis is renal disease. While there has not been sufficient data regarding the incidence of renal disease in patients with secondary syphilis, there have been several case reports on this topic. In one study, patients with kidney disease and syphilis who had undergone kidney biopsy were analyzed, which demonstrated that kidney injury most often manifested as membranous nephropathy (MN), but that acute interstitial nephritis, acute tubular injury, infection-associated glomerulonephritis, and IgA nephropathy were also identified [5].

Here, we present the case of a young woman who presented with a skin rash, was found to have nephrotic syndrome, and was ultimately diagnosed with secondary syphilis.

## Case presentation

A 35-year-old woman with a past medical history of iron deficiency anemia presented to the emergency department (ED) for shortness of breath, lower extremity edema, and a diffuse rash. The symptoms began two months prior to this admission, starting with a dry, flaky, nonpruritic rash. It began on the patient’s arm, with similar rashes later appearing on her palms, groin, lower extremities, and back. She was seen in the ED at that time and prescribed triamcinolone cream and permethrin for suspected scabies, yet the rash failed to improve. Six weeks later, the patient developed lower extremity edema and shortness of breath, which prompted her to return to the ED.

**Figure 1:**
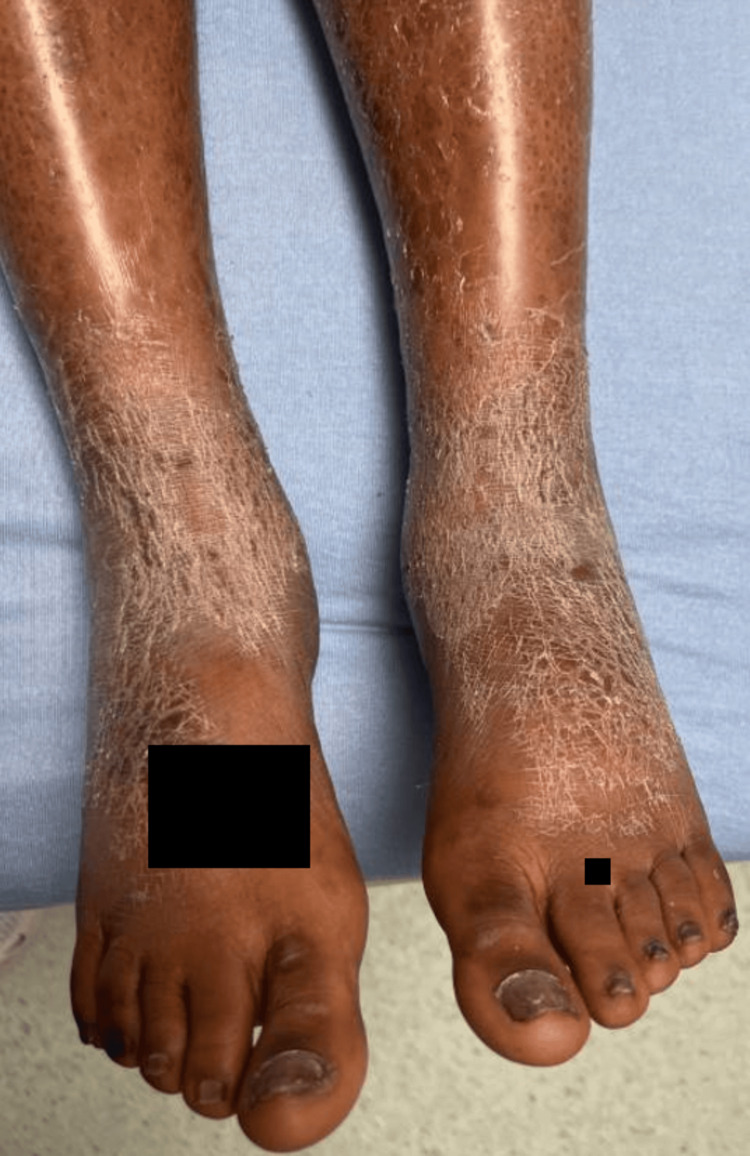
Dry rash observed on the patient’s bilateral ankles.

**Figure 2:**
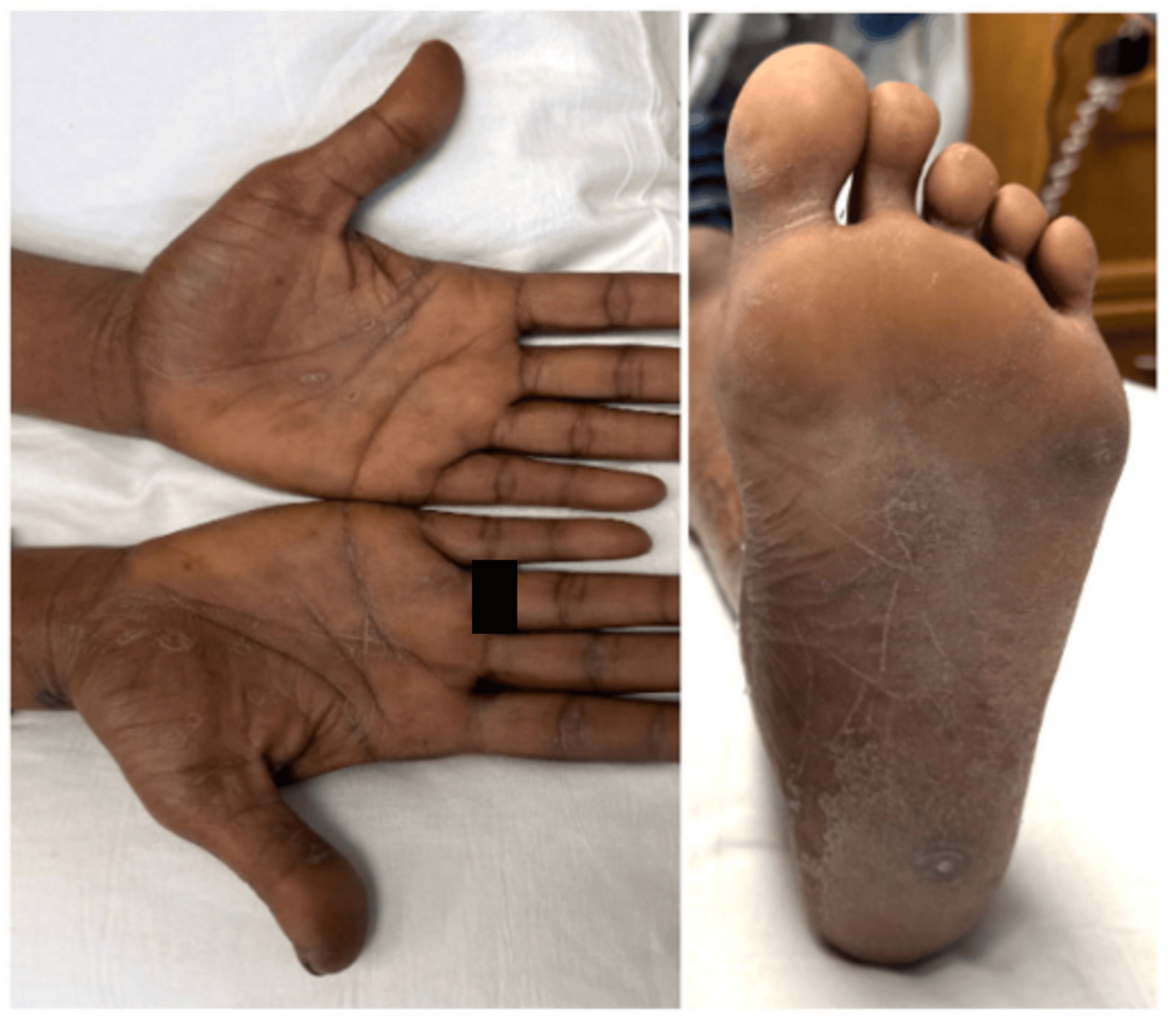
Lesions on the patient’s palms (left) and sole of the foot (right).

During the current admission, the patient’s blood pressure was 130/66 mmHg, heart rate was 75 beats per minute, oral temperature was 37.2°C (98.9°F), respiratory rate was 18 breaths per minute, BMI was 22.3, and SpO2 was 98% on room air. She was noted to have rough, flaky, dry skin on her bilateral ankles (Figure 1) as well as patchy areas of dry skin on other sections of her body. She also had symmetric, well-defined, scaly, and hyperkeratotic circular plaques on the palms and soles (Figure 2), with no vulvar or mucosal lesions. The patient also had bilateral pitting edema and diffuse tender lymphadenopathy, most notable in the inguinal region.

Serum creatinine on presentation was normal at 0.63 mg/dL. A urinalysis was notable for proteinuria at >1,000 mg/dL with a urine protein-to-creatinine ratio (UPCR) of greater than 2.23, indicative of nephrotic-range proteinuria. Urine microscopy was without casts, red blood cells, or white blood cells. Erythrocyte sedimentation rate (ESR) and C-reactive protein (CRP) were both elevated at >145mm/hr and 7.73 mg/dL, respectively. Lipid profile showed total cholesterol 147 mg/dL, low-density lipoprotein (LDL) 95 mg/dL, and triglycerides of 86 mg/dL. Liver studies demonstrated an albumin of 2.4 g/dL, with aspartate aminotransferase, alanine aminotransferase, alkaline phosphatase, and bilirubin all within normal range. These initial values with reference ranges are noted in Table 1.

**Table 1:** Initial laboratory values. ESR: erythrocyte sedimentation rate; CRP: C-reactive protein; LDL: low-density lipoprotein

Test	Reference Range	Results
Serum creatinine	0.51-0.95 mg/dL	0.63
Urine protein-to-creatinine ratio	<0.2 mg/mg	>2.23
ESR	0-20 mm/hr	>145
CRP	0.10-2.80 mg/L	7.73
Total cholesterol	100-199 mg/dL	147
LDL	0-99 mg/dL	95
Triglycerides	*≤*150 mg/dL	86
Albumin	3.8-4.9 g/dL	2.4
Aspartate aminotransferase	14-33 IU/L	33
Alanine aminotransferase	10-42 IU/L	53
Alkaline phosphatase	35-104 IU/L	386
Bilirubin	0.2-1.0 mg/dL	0.2

Given the wide differential diagnosis for nephrotic syndrome, workup for autoimmune, metabolic, and infectious etiologies was performed. This workup was negative for antinuclear antibody (ANA), anti-Smith antibody, anti-ribonucleoprotein antibody, anti-double-stranded deoxyribonucleic acid antibody (anti-dsDNA), anti-neutrophil cytoplasmic antibodies (ANCA), hepatitis B virus (HBV), hepatitis C virus (HCV), and human immunodeficiency virus (HIV). Complement component 3 (C3) and complement component 4 (C4) were normal. Serum treponemal pallidum antibody was positive, and the rapid plasma reagin (RPR) titer was 1:256, both a change from the patient’s negative syphilis screen during her delivery four months prior, indicative of a secondary syphilis infection. Hemoglobin A1c was 4.5%. This lab workup for nephrotic syndrome is further detailed in Table 2.

**Table 2:** Laboratory workup for nephrotic syndrome. ANA: antinuclear antibody; anti-dsDNA: anti-double-stranded DNA antibody; P-ANCA: perinuclear anti-neutrophil cytoplasmic antibody; c-ANCA: cytoplasmic anti-neutrophil cytoplasmic antibody; HCV: hepatitis C virus; HIV: human immunodeficiency virus; C3: complement component 3; C4: complement component 4; RPR: rapid plasma reagin; A1c: hemoglobin A1c

Test	Reference Range	Results
ANA	Negative	Negative
Anti-smith antibody	0.0-0.9 AI	<0.2
Anti-ribonucleoprotein antibody	0.0-0.9 AI	0.6
Anti-dsDNA	<5 IU/mL	1
P-ANCA titer	<1:20	<1:20
C-ANCA titer	<1:20	<1:20
Atypical P-ANCA titer	<1:20	<1:20
Hepatitis B surface antigen	Negative	Negative
HCV antibody	Negative	Negative
HIV 1/2 antigen/antibody screen	Negative	Negative
C3	90-180 mg/dL	143
C4	10-40 mg/dL	27
Treponema pallidum antibody	Nonreactive	Reactive
RPR titer	Nonreactive	1:256
A1c	4.0-6.0%	5.4%

**Figure 3:**
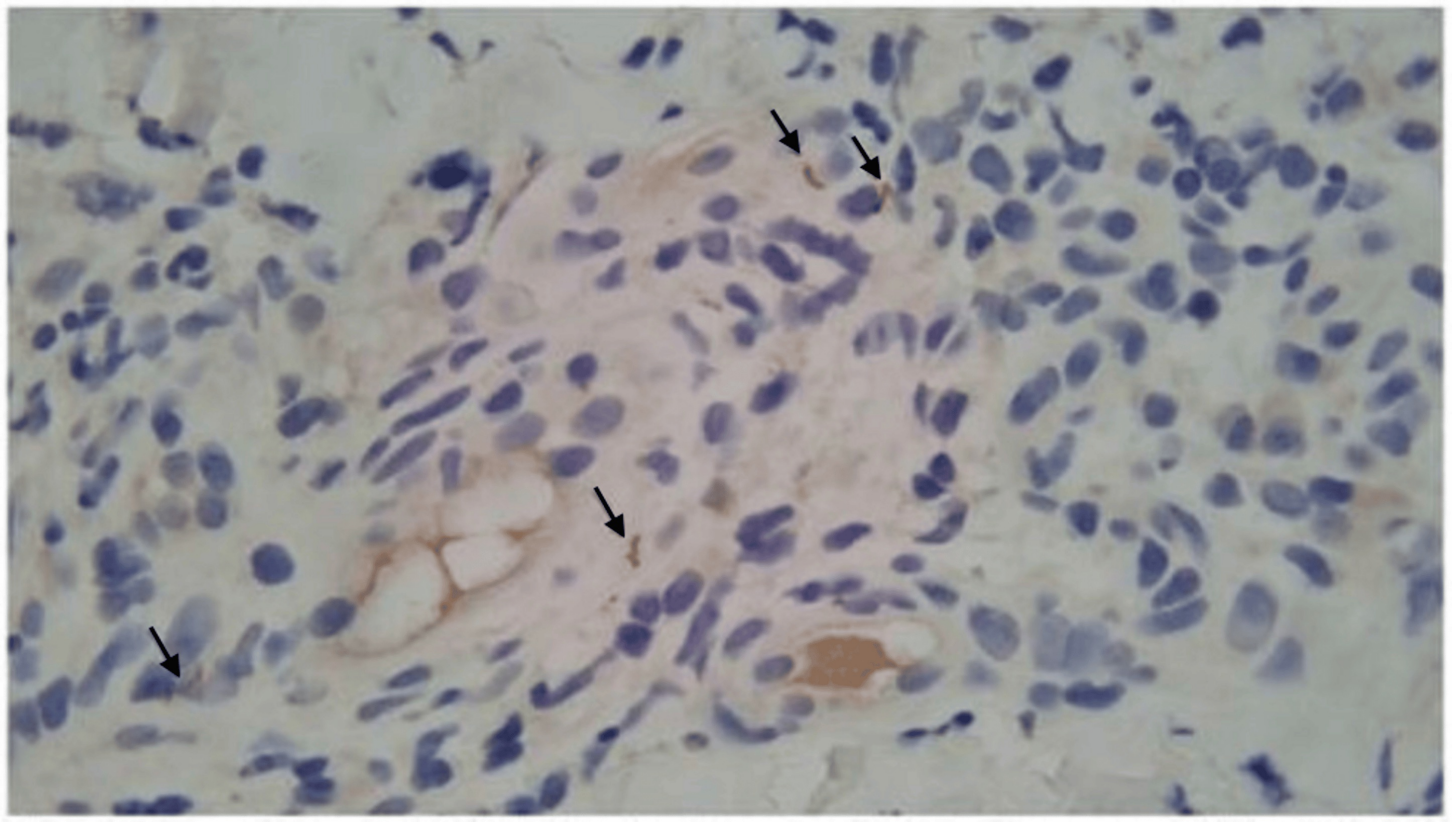
Treponema immunohistochemical stain highlights scattered treponemal spirochetes in the vascular endothelium in the papillary dermis (black arrows).

**Figure 4:**
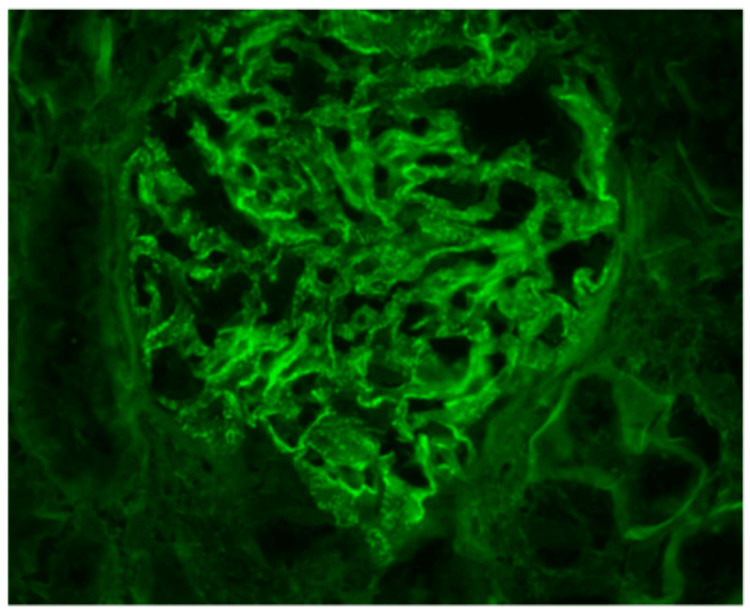
Fine granular staining of immunoglobulin G (IgG) shown in green on immunofluorescence at 400× magnification.

**Figure 5:**
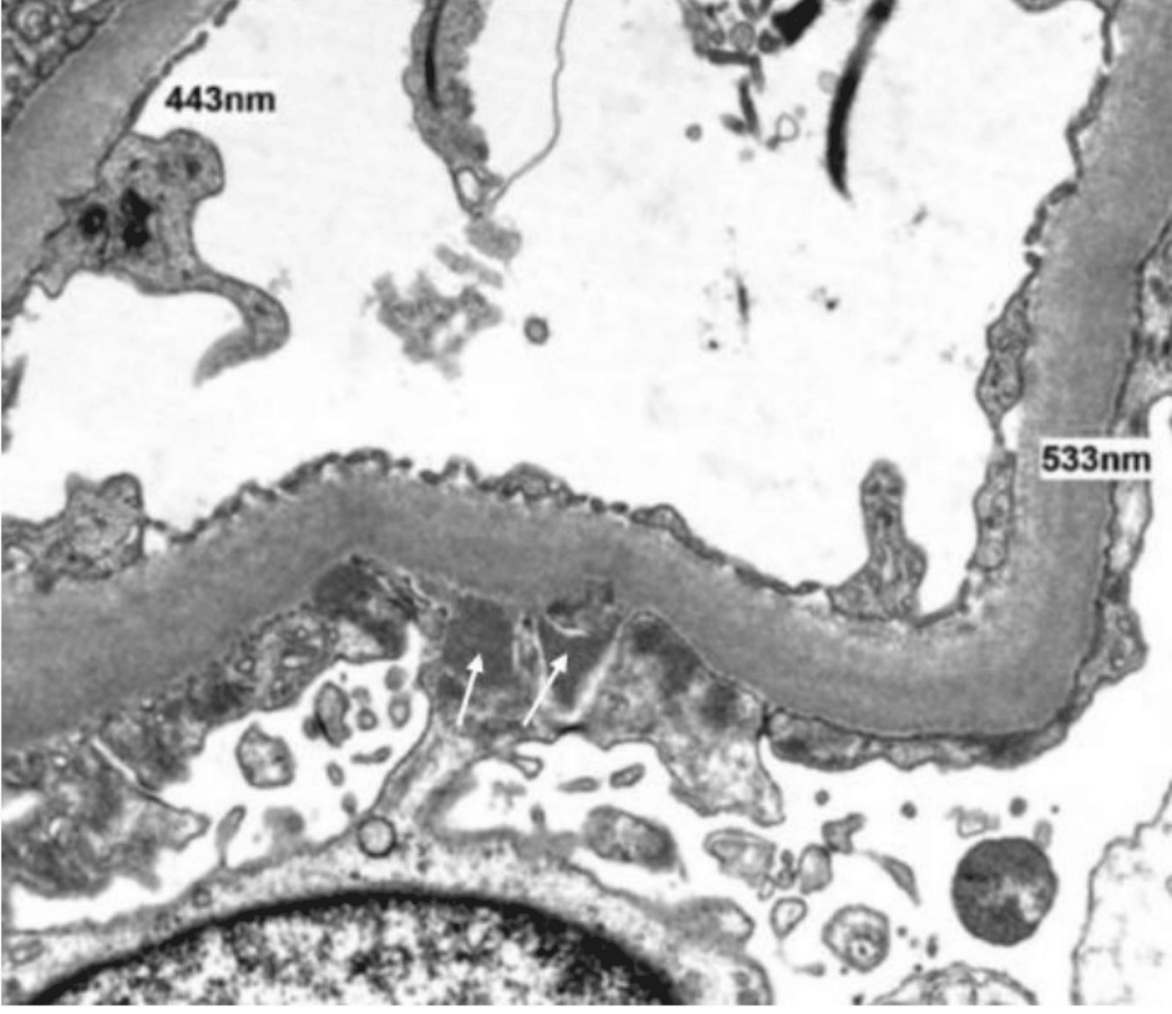
Subepithelial “humps” of immune-type electron-dense deposits (white arrows) seen on electron microscopy at 2000× magnification.

Computed tomography (CT) abdomen and pelvis, renal ultrasound, and echocardiogram were unremarkable. A skin punch biopsy of the ankle rash was negative for vasculitis, but did note spirochetes on a treponema histochemical stain (Figure 3). A percutaneous kidney biopsy revealed proliferation of mesangial cells, thickened basement membranes, effacement of epithelial foot processes, and granular immunofluorescent immunoglobulin G (IgG) and complement component 1q (C1q) deposition in subepithelial hump-like deposits, consistent with MN (Figures 4-5).

Based on the clinical course, serologic findings, and histopathological results, the patient was diagnosed with MN secondary to syphilis. She was treated with a single dose of penicillin G benzathine (2.4 million units) for secondary syphilis, as well as lisinopril for management of the new-onset nephrotic syndrome. Two days after treatment with penicillin, the patient’s UPCR improved to 0.61 mg/mg while serum creatinine remained unchanged at 0.6 mg/dL. After this treatment, the patient’s rash significantly improved. Two months following discharge, a repeat urinalysis was negative for proteinuria, and lymphadenopathy had resolved.

## Discussion

Despite the classic finding of palmar rash, typical of secondary syphilis, other confounding rashes on her ankles delayed the diagnosis of syphilis. As seen in this case, patients may present without ever having symptoms of primary syphilis, such as genital ulcers or chancres. Notwithstanding, the skin biopsy collected from the dry, flaky skin on the ankles stained positive for spirochetes. This exemplifies the disseminated nature of secondary syphilis. As spirochetes spread, the patient’s immune system mounts a robust response, creating immune complex deposition, and when affecting the kidneys, results in MN, manifesting as a phenotypic nephrotic syndrome.

Although the incidence of syphilis is increasing globally, renal involvement remains uncommon, with no exact data regarding its prevalence. The proposed mechanism is through basement membrane deposition of immune complexes, predominantly IgG and C1q, which leads to a diffuse glomerular capillary leak, non-selective proteinuria, and reduced renal function [6]. In this case, IgG and C1q deposition is observed, further differentiating it from autoimmune diseases like lupus, which present with deposition of IgA, IgG, IgM, C3, and C1q.

Nephrotic syndrome has a wide differential diagnosis, with the most common etiologies being diabetic, immunologic, malignant, infectious, and medication-related [7]. Within the infectious category, there are many viruses that have been found to be associated with nephropathy, including HIV, HBV, HCV, cytomegalovirus, and parvovirus [8]. Subclinical syphilis associated with MN is an extraordinarily rare occurrence [7]. Bacteria have also been known to damage the kidney, though more typically these present as a glomerulonephritis, such as in a *Streptococcus* infection [9]. These infections cause glomerular pathology through immune complex deposition with a variable inflammatory response that could result in a nephritis or a nephrosis.

Recent studies have further investigated the mechanism behind syphilis-induced MN, which recognized IgG deposition as the dominant subtype, as seen in this case. In addition to IgG, neuron-derived neurotrophic factor (NDNF), a novel target antigen, has now been identified as a more specific marker for nephrotic syndrome caused by syphilis [6]. Histopathology has, furthermore, revealed the localization of NDNF within subepithelial electron-dense deposits, confirming immune complex disease consisting of NDNF and IgG. NDNF decreases and disappears from the serum with successful antibiotic treatment of syphilis and nephrotic syndrome, making it another method of tracking treatment [10]. While this patient did not undergo testing for NDNF, it is important to consider it as it could be used in future cases as a confirmatory test for syphilis-induced MN in a patient who may have several other comorbidities.

Most patients with MN from secondary syphilis are thought to have excellent renal recovery after treatment with penicillin. A literature review of patients managed for this condition showed complete, often rapid renal recovery [11]. This was also observed in our patient who responded appropriately to the penicillin treatment.

## Conclusions

In a workup for nephrotic syndrome, patients are traditionally screened for autoimmune disease and several infectious causes. Even when the renal biopsy is indicative of MN, hepatitis and HIV are at the forefront of differential diagnoses, while syphilis can be forgotten despite having one of the widest-ranging clinical presentations of an illness. While this case has limitations, including a lack of NDNF testing, limited follow-up, and single case generalizability, it demonstrates that nephrotic syndrome from syphilis is easily treatable. Therefore, when clinicians encounter nephrotic syndrome, syphilis as an etiology should be considered, as it is curable if diagnosed and treated appropriately.
